# A Detailed Overview of Long-Term Outcomes in Severe Traumatic Brain Injury Eight Years Post-injury

**DOI:** 10.3389/fneur.2019.00120

**Published:** 2019-02-21

**Authors:** Alexis Ruet, Eléonore Bayen, Claire Jourdan, Idir Ghout, Layidé Meaude, Astrid Lalanne, Pascale Pradat-Diehl, Gaëlle Nelson, James Charanton, Philippe Aegerter, Claire Vallat-Azouvi, Philippe Azouvi

**Affiliations:** ^1^Physical Medicine and Rehabilitation Department, CHRU, Caen, France; ^2^Laboratoire de Recherches Cliniques et en Santé publique sur les Handicaps Psychiques, Cognitifs et Moteurs (HANDIReSP, EA4047), Université de Versailles Saint-Quentin, Montigny-Le-Bretonneux, France; ^3^EPHE, INSERM, U1077, Neuropsychologie et Imagerie de la Mémoire Humaine, Caen, France; ^4^Physical Medicine and Rehabilitation Department, Pitie-Salpetriere Hospital, APHP, Paris, France; ^5^Sorbonne Université GRC18, Paris, France; ^6^Physical Medicine and Rehabilitation Department, Lapeyronie Hospital, CHRU, Montpellier, France; ^7^Unité de Recherche Clinique Paris Ile-de-France Ouest, Ambroise Paré Hospital, APHP, Boulogne, France; ^8^Physical Medicine and Rehabilitation Department, APHP, Raymond-Poincaré Hospital, Garches, France; ^9^Laboratoire d'Imagerie Biomedicale Inserm U1146, Sorbonne Université GRC18, Paris, France; ^10^Regional Reference Center for Bain Injury in the Parisan Area, CRFTC, Paris, France; ^11^Antenne UEROS-SAMSAH92-UGECAM IDF, Hôpital Raymond Poincaré, Garches, France; ^12^Laboratoire de Psychopathologie et Neuropsychologie, EA 2027, Université Paris 8, Saint-Denis, France

**Keywords:** traumatic brain injury, outcome, longitudinal study, adult, independence, return to work

## Abstract

**Background and aims:** Severe traumatic brain injury is a leading cause of acquired persistent disabilities, and represents an important health and economic burden. However, the determinants of long-term outcome have rarely been systematically studied in a prospective longitudinal study of a homogeneous group of patients suffering exclusively from severe TBI

**Methods:** Prospective observational study of an inception cohort of adult patients with severe traumatic brain injury in the Parisian area (PariS-TBI). Outcome was assessed with face-to-face interview 8 years after Traumatic Brain Injury, focusing on impairments, activity limitations, and participation restriction.

**Results:** Five hundred and four patients were included between 2005 and 2007. At 8-year follow-up, 261 patients were deceased, 128 were lost to follow-up, 22 refused to participate, and 86 were finally evaluated. Age, gender, initial injury severity did not significantly differ between evaluated patients and lost to follow-up, but the latter were more frequently students or unemployed. Mean age was 41.9 (*SD* 13.6), 79% were male, median initial Glasgow Coma Scale Score was 6. The most frequent somatic complaints concerned balance (47.5%), motricity (31%), and headaches (36%), but these were less frequent than cognitive complaints (Memory 71%, Slowness 68%, Concentration 67%). According to the Hospital Anxiety and Depression Scale (HADS), 25 % had a score >8 for anxiety and 23.7% for depression. According to the Extended Glasgow Outcome Scale, 19.8% remained severely disabled, 46.5% moderately disabled, 33.7% had a good recovery. Older age, longer education duration, lower functional status upon intensive care discharge, and more severe 8-year dysexecutive problems were significantly associated with a lower Extended Glasgow Outcome Scale score in multivariable analysis. At 8 years, 48.7% of patients were employed in a productive job. Of those, 38% declared a salary loss since traumatic brain injury. Unemployment was significantly associated with lower 1-year GOSE score and more severe 8-year dysexecutive problems.

**Conclusions:** These results from an inception cohort study highlight the fact that long-term outcome after severe TBI is determined by a complex combination of injury-related, demographic and neuropsychological factors. Long after the injury, persisting impairments still interfere with social integration, and participation.

## Introduction

Traumatic brain injury (TBI) is a leading cause of persistent disability worldwide. The likelihood of suffering disability increases with severity of TBI. In European countries, an overall incidence of 262 hospitalizations/100,000 inhabitants per year was derived from a meta-analysis (1). About 10% of TBI are considered as severe. The weighted average mortality for severe traumatic brain injury was 39%, and for an unfavorable outcome on the Glasgow Outcome Scale was 60% according to a 2012 meta-analysis (2). TBI often occurs in young adults who will live decades with a variety of cognitive, emotional, physical and sensory disabilities (3). Participation limitations have been described in numerous studies focusing on TBI outcome (4). In the majority of previous studies, outcome, and prognostic factors were assessed within the first 5 years after TBI (5, 6). It is admitted that the major part of the functional improvement is made in the first year (7, 8). However, some studies found a long-term improvement of independence or a relative stability (3, 9). These studies included patients from rehabilitation centers and might not be representative samples of all TBI patients as previous research found that severe TBI patients were not systematically referred to in-patient rehabilitation units (10, 11). Other studies, described the longitudinal outcome of severe TBI patients included since acute care but with relatively small samples (12, 13). Hence, there is a lack of knowledge about the long-term outcome (after 5 years) in homogeneous, consecutively admitted from acute care, and exclusively severe TBI patients.

Previous studies have shown that long-term outcome in term of global functioning of participation in a paid productive activity was determined by multiple factors, some of which were socio-demographic, such as age and sex, others were related to the severity of TBI (4, 6). The aim of the present study was to give a comprehensive picture of long-term (8-year) outcome in an homogeneous sample of patients with severe TBI and to assess its determinants or related factors, in line with the previous reports on this cohort (11, 14–19).

## Materials and Methods

### Participants, PariS-TBI Study

Adults (aged more than 15 years) with severe traumatic brain injury (Glasgow Coma Scale score ≤8 before the hospital admission in absence of other cause of coma) in the Parisian area were recruited consecutively by mobile emergency services from 2005 to 2007 (20). A total of 504 patients were included. Main causes of injury were road traffic accidents (52%) and falls (34%). Pre-injury characteristics including gender, age, education duration, professional status as well as a history of alcohol abuse were documented from medical records and from information provided by relatives. The initial assessment included an assessment of disability at the intensive care unit (ICU) discharge with the Glasgow Outcome Scale (GOS) (21). Patients were followed-up at one, 4 and 8 years post-injury. The 1-year assessment was undertaken by a trained neuropsychologist by telephone interview with patients and their relatives. Four and Eight year outcome were documented by a face to face interview conducted by trained neuropsychologists with patients and their relatives. During the one, 4 and 8 year evaluations, a standardized questionnaire was used and various data were collected about home situation, marital status, work or study status, functional status, disabilities, and complaints. Inclusion criteria at the acute stage and data on 1 and 4-year outcome of the PariS-TBI study have been extensively reported in previous publications (11, 14–18, 22, 23). In the present study, we focused on patients who were evaluated at 8 years post-TBI.

There were 86 patients who attended 8-year follow-up, 268 patients were deceased (247 during the acute stage), 128 patients were lost to follow-up and 22 refused to participate. Sixty-eight (79%) out of the 86 evaluated patients were men, with a mean age at the time of TBI of 34 years (standard deviation [SD], 13.7), mean age at the time of evaluation of 41.9 years (*SD*, 13.6) and mean education duration 12.2 years (*SD*, 3.2). The initial Glasgow Coma Scale (GCS) score was three or four for 25.9% of the sample, five or six for 34.1%, seven or eight for 40%. The mean time to follow command was 12.4 days (SD, 10.6) and the mean length of stay in the intensive care unit was 28.8 days (SD, 23.8). The mean time since injury was 98.5 months (SD, 8.65). Four of the 86 patients refused to complete the totality of the questionnaire. Regarding return to work assessment, patients who were retired at the time of TBI or over 64 years old at 8 years were excluded resulting in a 76 patients sample.

### Assessment

At 8 years post-TBI, a clinical evaluation and the standardized questionnaire assessed the independence in simple and complex daily living activities. For the study, we created a questionnaire to assess the neurological and somatic impairments. Patients were asked the following question “What are the physical difficulties you have because of head trauma?.” Eleven of the most frequent deficiencies reported in previous studies were proposed with the possibility of classifying them as “none,” “moderate,” “severe.” Cognitive and behavioral complaints were investigated with the Brain Injury Complaint Questionnaire (BICoQ), after the following explanation “We will ask you questions about the problems you face in your everyday life since the TBI.” Twenty-five closed questions were given addressing frequently reported cognitive and behavioral complaints (19, 24). The same questions were asked to their relatives. Patients were asked about the recurrence of TBI and whether they suffered epilepsy or not.

The structured interview was developed for the study to assess impairments, activities, and participation according to the International Classification of Functioning, Disability, and Health framework (25). The global outcome was evaluated with the French version of the Glasgow Outcome Scale-Extended (GOSE) (20, 26). This frequently used rate scale allows classifying people in eight categories ranging from death to upper good recovery.

The Dysexecutive Questionnaire (DEX) was completed by the patient and their relative to assess executive dysfunctions in daily life (27). This is a 20-item questionnaire covering four broad areas of likely changes: emotional or personality changes, motivational changes, behavioral changes and cognitive changes. Each item is scored on a five-point (0–4) Likert scale (ranging from never to very often). The DEX is a multidetermined sensitive questionnaire to detect everyday life difficulties in patients with severe TBI at a chronic stage (23). Mood impairments were measured by the Hospital Anxiety and Depression Scale (HADS) (28), which has two subscores, for anxiety and depression, both ranging from 0 to 21 (highest anxiety or depression).

Patients were asked about the continuation or resumption of studies after TBI and working situation. Post-TBI difficulties at work were explored by a dedicated questionnaire which was developed by a group of experts and routinely used in a vocational rehabilitation unit. Self-perception and the consequences of the difficulties were assessed by the questionnaire and the responses, concerning twenty-two difficulties at work, were binary. Patients were then asked to assess the frequency and intensity of their difficulties at work on a four-point scale. Finally, patients were asked to estimate their perceptions of their peer's consciousness and tolerance of their difficulties at work, on a scale from zero to ten.

### Ethics Approval Statement

In accordance with French legislation, patients and their relatives were informed about their initial inclusion in the database. Informed written consent from participants (or their legal representatives) was obtained before each study assessment. Furthermore, before the assessments at each study stage, approval was granted from Commissions which enforce research database legislation in France, and the local Ethical Committee (Comité de Protection des Personnes, CPP XI). The study was recorded in the ClinicalTrials.gov database in January 2014 (identifier: NCT02050633).

### Statistical Analysis

Preinjury sociodemographic factors, injury-related factors, post-injury factors were described using means, standard deviations, minima and maxima for continuous variables. Median and interquartile range were used to describe numerical variables in small samples. Categorical variables were described using counts and percentages. Data were sometimes incomplete because some individuals did not provide answers to all questionnaires. In case of missing data, percentages were based on the number of subjects who answered the given questionnaire.

For univariate comparisons between employed and unemployed subjects at 8 years, we used a two-sided statistical analysis and a 5% significance level. Student's *t*-tests were used for continuous variables, Chi2 tests were used for categorical variables. When Chi2 results showed a dependent relation between the studied variables, the adjusted standardized residuals were calculated to assess the statically significant differences among cells of the contingency table (29, 30). For univariate analysis of 8-year GOSE score, an ordinal regression with cumulative link model was computed with each independent variable if proportionality assumption of the odds was met and a Spearman's correlation was calculated if not. Because of multiple comparisons in univariate analysis of GOSE and return to work related factors and associated type I error inflation, results were given with both *p*-values and corrected *p*-values according to Holm (31). For multivariable analysis of 8-years GOSE score, an ordinal regression with cumulative link model was computed and proportionality assumption of the odds was verified. We computed a two-step analysis with a first model including sociodemographic and injury-related variable and a second model including sociodemographic, injury related and post-injury factors. Independent variables were chosen if they were statistically significant in the univariate analysis. Although non-significant in the univariable analysis, age was kept in the multivariable analysis because previous studies found an important association between older age and poor functional outcome (4). For the first model, a stepwise selection of independent variable was made starting with the full model and iteratively removing the least contributive predictors, and stopping when having a model where all predictors were statistically significant. In the second model, although statistically significant in the univariate analysis, HADS depression and total scores were not included because the proportional odds assumption was not met for these variables.

## Results

### Comparison Between Evaluated Patients and Lost to Follow up or Refusal to Participate

The univariate analysis of the comparison between evaluated patients and lost to follow up or refusal to participate is presented in Table 1. Evaluated and non-evaluated patients were statistically significantly different regarding preinjury occupational level (Chi^2^ = 23.637, df = 4, *p*-value < 0.001). Adjusted standardized residuals were only >±2 for the “white collar” occupational class. White collar patients were significantly overrepresented amongst evaluated patients. There were no other between-group differences (particularly initial injury severity was not significantly different in the two groups). There was a trend for unevaluated patients to have shorter education duration and to be unemployed before TBI without Holm *p*-value correction.

**Table 1:** Univariate comparison of evaluated and non-evaluated patients.

	**Non-evaluated *n* = 150**		**Evaluated *n* = 86**	**Missing data**	**p (chi2) or p (student)**	Corrected^*^
**Patient characteristics**	**Mean ± SD [minimum; maximum] or count (%)**	**Missing data**	**Mean ± SD [minimum; maximum] or count (%)**			**p (chi2) or p (student)**
Gender		0 (0%)		0 (0%)	0.801	1
Female	28 (18.7%)		18 (20.9%)			
Male	122 (81.3%)		68 (79.1%)			
Age at time of TBI (years)	33.5 ± 15.9 [15.2–82.7]	2 (1.3 %)	34.1 ± 13.7 [15.4–74.8]	0 (0 %)	0.753	1
Education duration (years)	11 ± 2.6[5-18]	58 (38.7 %)	12.2 ± 3.2 [6-20]	8 (9.3 %)	0.008	0.099
Occupational class		32 (21.3%)		4 (4.7%)	< 0.001	0.001
Blue collar	52 (44.1%)		33 (40.2%)			
White collar	5 (4.2%)		22 (26.8%)			
Retired	11 (9.3%)		4 (4.9%)			
Student	30 (25.4%)		17 (20.7%)			
Unemployed	20 (16.9%)		6 (7.3%)			
Employment preinjury		32 (21.3%)		4 (4.7%)	0.006	0.115
Yes	68 (57.6%)		59 (72%)			
No	50 (42.4%)		20 (24.4%)			
Living alone before TBI		4 (2.7%)		0 (0%)	0.257	1
No	113 (77.4%)		60 (69.8%)			
Yes	33 (22.6%)		26 (30.2%)			
Alcohol addiction before TBI		10 (6.7%)		5 (5.8%)	0.309	1
No	118 (84.3%)		73 (90.1%)			
Yes	22 (15.7%)		8 (9.9%)			
Initial GCS		7 (4.7%)		1 (1.2%)	0.942	1
3–4	36 (25.2%)		22 (25.9%)			
5–6	52 (36.4%)		29 (34.1%)			
7–8	55 (38.5%)		34 (40%)			
Duration of coma (days)	8.8 ± 7.7 [0–50]	18 (12 %)	9.6 ± 6.2 [0–24]	15 (17.4 %)	0.406	1
Time to follow command (days)	11.8 ± 11.9 [0–81]	22 (14.7 %)	12.4 ± 10.6 [0–56]	20 (23.3 %)	0.737	1
Length of stay in ICU (days)	24.6 ± 19.7 [2-134]	1 (0.7 %)	28.8 ± 23.8 [2–131]	0 (0 %)	0.171	1
GOS at ICU discharge	3.8 ± 0.9 [2–5]	20 (13.3 %)	3.8 ± 0.8 [2–5]	12 (14 %)	0.857	1

*GCS, Glasgow Coma Scale; ICU, Intensive Care Unit; GOS, Glasgow Outcome Scale*.

* *Corrected p-value according to Holm*.

### Impairments, Activity Limitations, Global Outcome, and Living Situation

**Figure 1:**
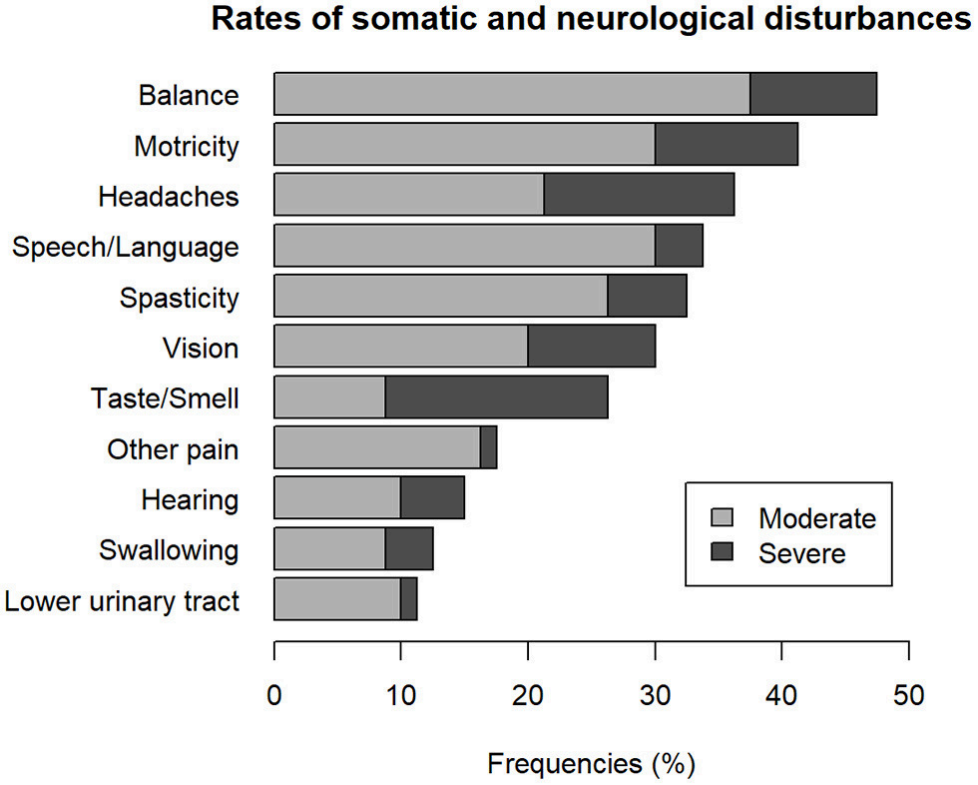
Frequency of somatic and neurological complaints.

**Figure 2:**
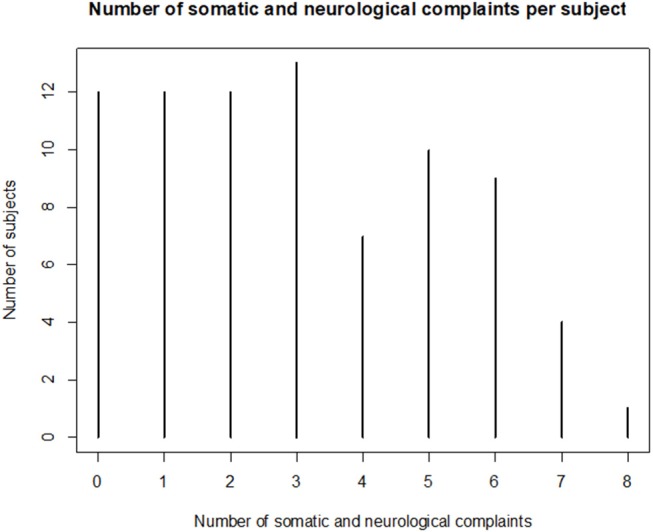
Number of somatic and neurological complaints per subject 8 years after severe traumatic brain injury.

**Figure 3:**
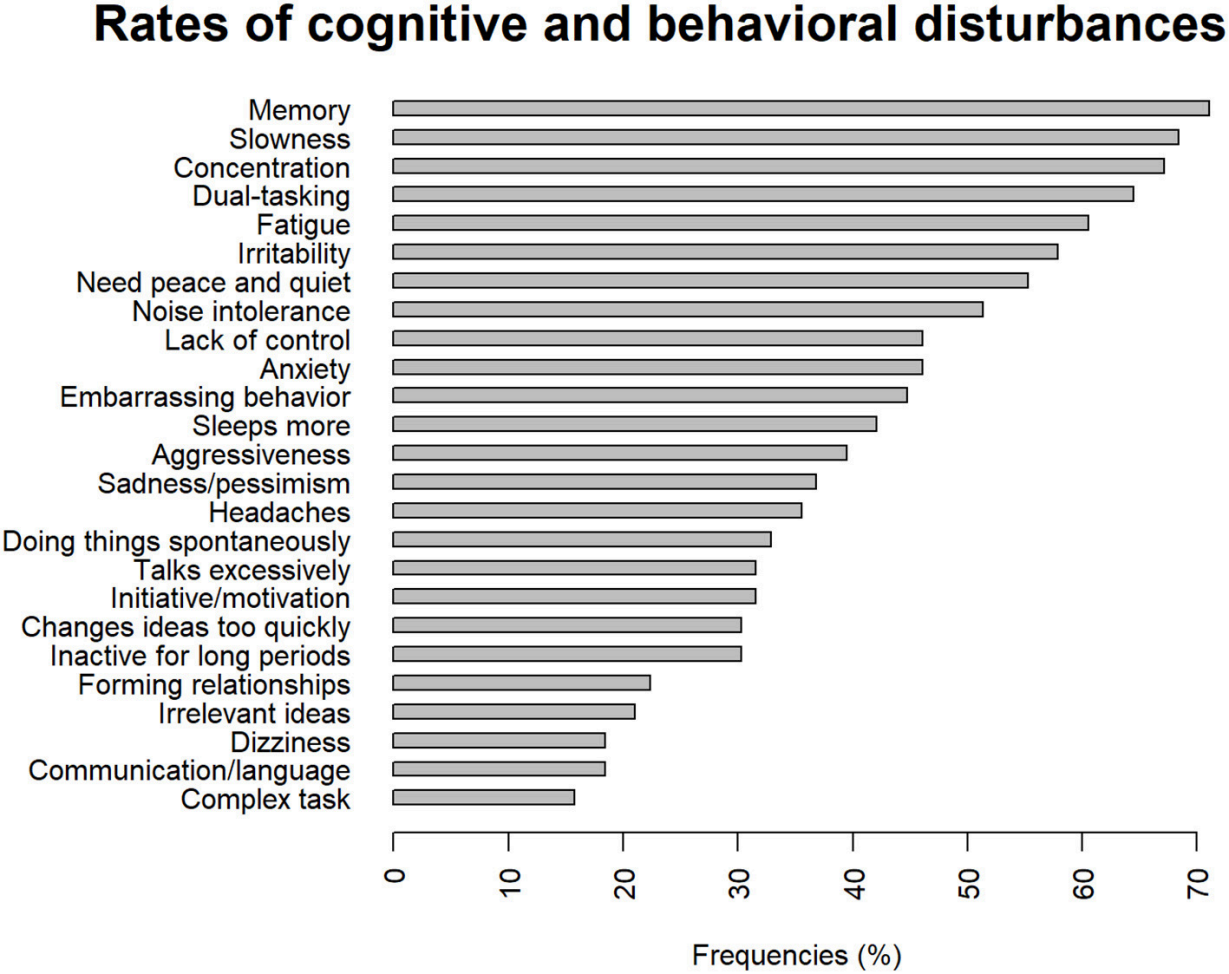
Frequencies of cognitive and behavioral disturbances 8 years after severe Traumatic Brain Injury.

**Figure 4:**
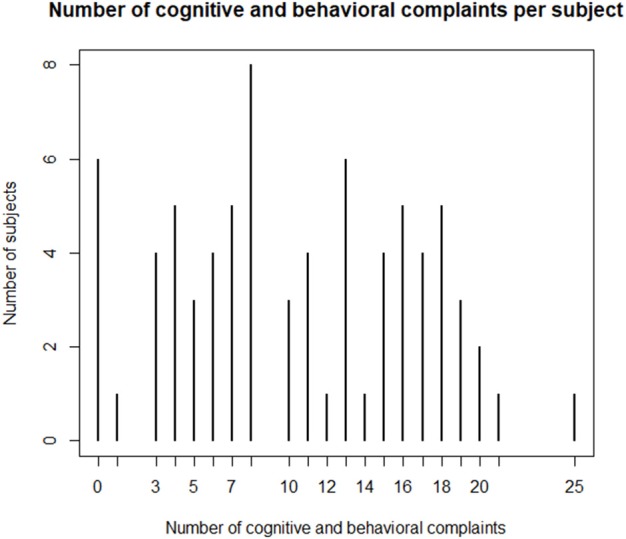
Number of somatic and behavioral complaints 8 years after severe Traumatic Brain Injury.

The frequency of somatic and neurological complaints of the 80 patients who completed the questionnaire is shown in Figure 1. The three most frequent somatic and neurological complaints were balance, motricity and headaches. Taste and smell complaints were the most frequently reported as severe by 17.5% of the sample. The sample had three somatic or neurological complaints at mean (*SD*, 2.2) and 15% did not have any complaint at all. The number of somatic or neurological complaint per subject is shown in Figure 2. The frequency of cognitive and behavioral complaints of the 76 patients who completed the questionnaire is shown in Figure 3. Eight complaints were reported by more than a half of the sample: noise intolerance (51.3%), need peace and quiet (55.3%), irritability (57. 9%), fatigue (60.5%), dual-tasking (64.5%), concentration (67.1%), slowness (68.4%), memory failures (71.1%). The mean number of cognitive and behavioral complaints was 10.4 (*SD*, 6.2) and only 7.9% of the sample reported none of the 25 complaints of the questionnaire. The number of complaints per subjects is shown in Figure 4.

None of the patients suffered another TBI in 8 years. Nine patients out of 82 (11%) declared having suffered seizure since TBI but only one had seizures during the past year.

Mean HADS scores were 6.2 (*SD*, 4.6) for anxiety and 5.5 (*SD*, 4.6) for depression. According to the previously defined cut-off score of 8 points for these subscales, 8 subjects (10.5%) had an anxiety disorder and 7 (9.2%) had depression, 11 (14.5%) had both anxiety disorder and depression.

**Figure 5:**
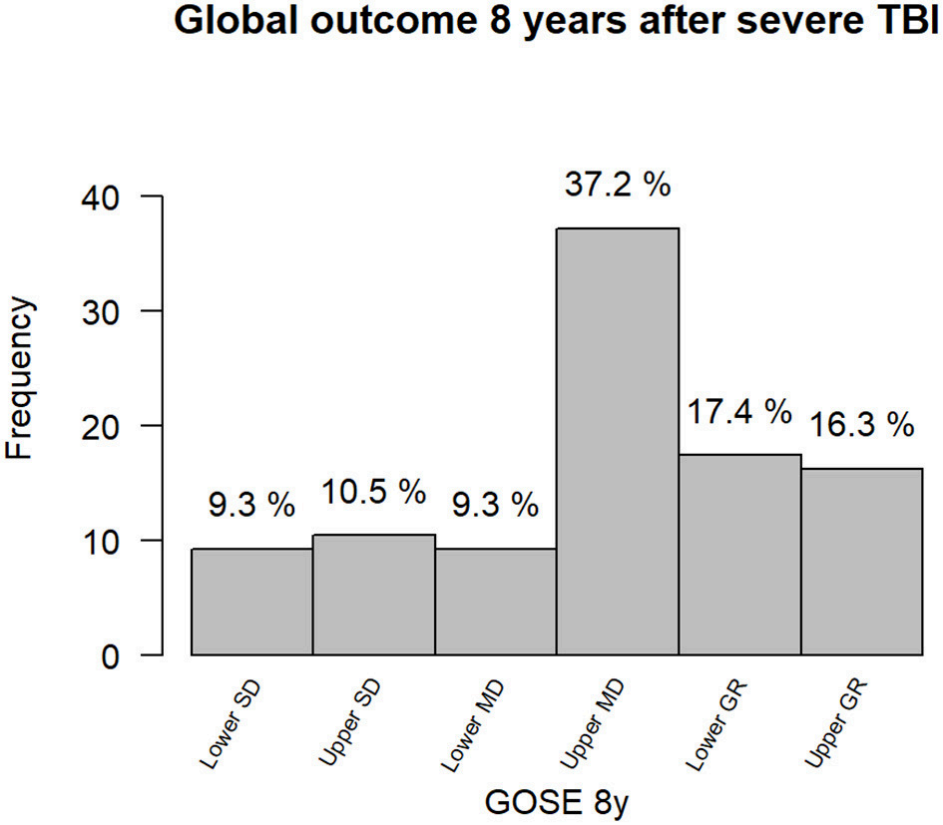
GOSE 8y: Glasgow Outcome Scale Score Extended at 8 years. SD, Severe Disability; MD, Moderate Disability; GR, Good Recovery.

Global outcome based on GOSE scores is shown in Figure 5. The majority of patients (37%) fell in the upper Moderate Disability category.

Univariate analysis of variable associated with GOSE score at 8 years is shown in Table 2. A longer education, a shorter length of stay in intensive care unit, a higher GOS score upon intensive care discharge, a higher GOSE score at 1 year were associated with better outcome (i.e., a higher 8-year GOSE score). HADS depression and total scores as well as DEX total scores rated by patients or relatives were significantly higher amongst subjects with lower GOSE score after correction of the *p*-value according to Holm. There was a trend for higher initial GCS score to be associated with higher GOSE score at 8 years before *p*-value correction. On multivariable analysis (Table 3), the first model including sociodemographic and injury-related factors revealed that older age, shorter education duration, longer length of stay in ICU and lower GOS score at ICU discharge were associated with poorer global outcome. The initial GCS score was not kept by the stepwise selection. All variables of the first model except length of stay in ICU were still significantly associated with GOSE score after adding DEX score rated by patients. A lower DEX score was associated with a poorer global outcome.

**Table 2:** Years GOSE univariate analysis.

	**OR [95% CI] or spearman's rho**	***p*-value**	Corrected^*^p-value
Age (years) (*n* = 86)	0.977 [0.949–1.004]	0.101	0.909
Gender male (*n* = 86)	0.976 [0.397–2.40]	0.958	1
Education duration (years) (*n* = 78)	1.23 [1.07–1.41]	0.003	0.051
Occupational Class pre-injury (*n* = 82) (ref. blue collar)			
White collar	1.43 [0.537–3.87]	0.472	1
Retired	1.28 [0.163–9.91]	0.81	1
Student	2.92 [1.002–8.69]	0.051	0.56
Unemployed	1.00 [0.235–4.31]	0.998	1
White collar	1.43 [0.537–3.87]	0.472	1
Employed pre-injury (*n* = 82)	0.583 [0.250–1.34]	0.206	1
Living alone pre-injury (*n* = 86)	1.08 [0.467–2.52]	0.854	1
Alcohol abuse (*n* = 81)	0.738 [0.209–2.66]	0.637	1
GCS (*n* = 85)	3.74 [1.40–10.2]	0.009	0.126
Duration of coma (days) (*n* = 71)	0.953 [0.886–1.02]	0.193	1
Time to follow command (days) (*n* = 66)	0.958 [0.915–1.003]	0.068	0.681
Length of stay in ICU (days) (*n* = 86)	0.972 [0.955–0.989]	0.002	0.027
GOS at ICU discharge (*n* = 74)	2.01 [1.20–3.43]	0.009	0.126
GOSE at 1 year (*n* = 55)	5.16 [3.01–9.62]	< 0.001	< 0.001
HAD anxiety at 8 years (*n* = 76)	0.884 [0.805–0.969]	0.009	0.126
HAD depression at 8 years (*n* = 76)	−0.498	< 0.001	< 0.001
HAD total at 8 years (*n* = 76)	−0.428	< 0.001	0.002
DEX score at 8 years (patients) (*n* = 76)	0.954 [0.924–0.983]	0.003	0.048
DEX score at 8 years (relatives) (*n* = 47)	0.946 [0.910–0.979]	0.003	0.045

TBI, Traumatic Brain Injury; GCS, Glasgow Coma Scale; GOS, Glasgow Outcome Scale; ICU, Intensive Care Unit; GOSE, Glasgow Outcome Scale—Extended; HADS, Hospital Anxiety Depression Scale; DEX, Dysexecutive Questionnaire; OR, Odds Ratio of falling into a upper level of GOSE associated with a one-unit increase of the independent variable.

* *Corrected p-value according to Holm*.

**Table 3:** Eight-years GOSE score multivariable analysis.

**Variable**	**Model 1**		**Model 2**	
	Sociodemographic and injury related factors (*n* = 60) OR [95% CI]	*p*-value	Sociodemographic, injury related and post injury factors (*n* = 60) OR [95% CI]	*p*-value
Age (years)	0.94 [0.9–0.98]	0.002	0.96 [0.92–1.00]	0.03
Education (years)	1.38 [1.18–1.63]	0.0001	1.3 [1.09–1.56]	0.004
Length of stay ICU	0.97 [0.95–0.998]	0.03	0.98 [0.95–1.01]	0.3
GOS score at ICU discharged	2.19 [1.22–4.02]	0.04	2.11 [1.11–4.08]	0.02
DEX score (patient) at 8 years	–	−	0.96 [0.92–0.99]	0.02

*GOSE, Glasgow Outcome Scale—Extended; GOS, Glasgow Outcome Scale; ICU, Intensive Care Unit; DEX, Dysexecutive Questionnaire; OR, Odds Ratio of falling into a upper level of GOSE associated with a one-unit increase of the independent variable*.

**Figure 6:**
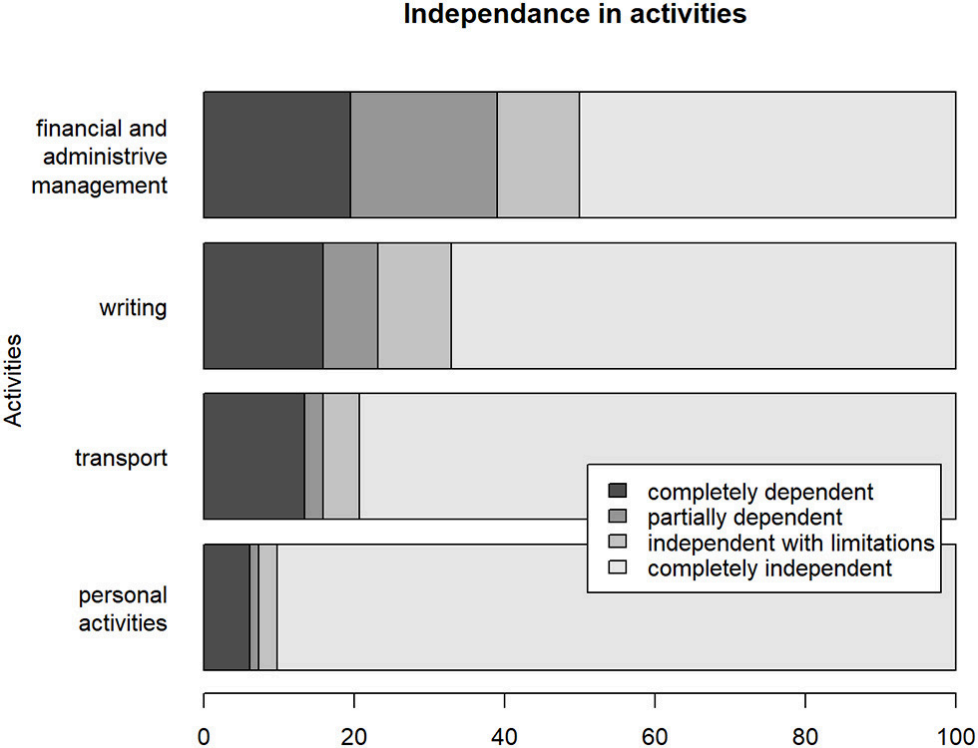
Writing, Write a letter; Transport, Taking public transports; Personal activities, grooming, dressing, moving at home, using the bathroom.

The majority of subjects (90.2%) declared to be independent for dressing, grooming, moving inside the home, using the bathroom, 79.3% for taking public transport, 67.1% for writing a letter, 50% for financial and administrative management. Figure 6 shows subjects independence or need for support in these activities. Most of the patients (51.2%) declared to be able to drive without limitation, 12.2% only on short distances travels, 36.6% did not drive motor vehicles. Only 40.4% of the subjects who resumed driving revalidated their driving license with an approved practitioner for capacity for driving.

Nearly half of the patients (49.4%) were in a relationship, 22.9% had children at home, 3.6% were single with children at home. Most of the patients (77.1%) declared living in their own home, 18.1% lived in a relative's home, 3.6% were living in an institution, one (1.2%) was still hospitalized since the TBI. Home Accessibility Modifications were made for 16.9% of patients and they all received funding to make the modification.

### Education and Work

Six out of the 17 students at the time of the injury continued their education. These six patients had a job at 8-year follow-up. Of the 11 students who did not continue education, seven did not work at 8 years.

Eight years after the injury, 37 subjects (45.1% of the 82 evaluated patients and 48.7% of patients aged under 65) had a job. Only one patient worked in a sheltered workshop. Seventeen patients (41.5% of workers), declared that their job changed after TBI. This job modification corresponded to an occupational reclassification for 10 subjects (58.8%) and to a modification of tasks in the same kind of job for seven (41.2%). Twelve patients (70.6%) had changed employer. In comparison to pre-injury, among the 37 employed patients, nine (24.3%) decreased their working time, 25 (67.6%) remained the same and 3 (8.1%) increased their working time. Incomes had decreased for 14 subjects (37.8%), had remained stable for 19 (51.4%) and increased for 4 (10.8%). Regarding their responsibilities at work, two subjects (5.4%) declared an increase, 29 (78.4%) had not reported a change, 6 (16.2%) declared a decrease. Most of the patients (n = 27, 73%) worked 80% to full time, eight (21.6%) worked half time to 79%, two (5.4%) worked less than half-time. Twenty-six subjects declared to plan a career development in the future. On average, subjects resumed work 27.8 months (*SD*, 27) after TBI. Seventeen patients (45.9%) followed vocational training which was a paid training course for 10 of them (58.8%). Six patients followed a vocational rehabilitation program.

**Figure 7:**
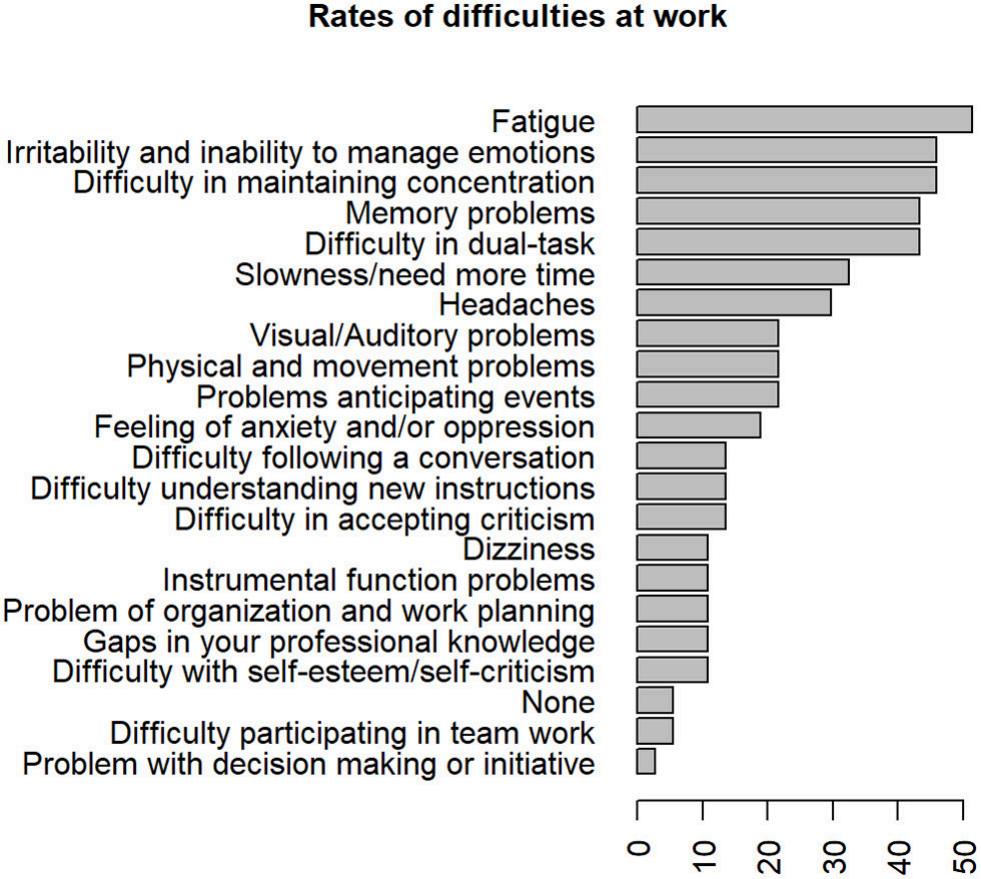
Frequencies of reported difficulties at work 8 years after severe Traumatic Brain Injury.

Subjects who had a job were asked for their difficulties at work. The main complaints were fatigue (*n* = 19, 51.4%), irritability and inability to manage emotions (*n* = 17, 45.9%), difficulty in maintaining concentration (*n* = 17, 45.9%), difficulty in dual-tasks (*n* = 16, 43.2%), memory problems (*n* = 16, 43.2%). Rates of difficulties at work are shown in Figure 7. The median number of difficulties at work was five (interquartile range, 5). Only two of the 37 subjects declared no difficulties. These difficulties were considered constant for 6 subjects out of 35 (17.1%), frequent for 11 (31.4%), occasional for 18 (51,5%) and they answered that it disturbed work “a lot” for 8.6%, “a little” for 42.9%, “not at all” for 48.6%. Finally, on the 37 workers, 40.5% answered that their quality of life had improved “enormously,” 40.5% “a lot,” 16.2% “a little,” and 2.7 % “not at all” since they returned to work.

Ten subjects out of the 82 evaluated patients (12.2%), returned to work after the injury and then quit. Three of them (30%), declared a job change and two (20%) a modification of tasks in the same kind of job. Four of these 10 patients had changed employer. These ten subjects returned to work at median 24 months after TBI (interquartile range, 9). They quit at median 21 months (interquartile range, 19.5) later. Job loss was related to TBI according to five of these patients. Three subjects declared they quit work because of difficulties at work, one retired, another stopped work for a professional training, three were at the end of their employment contract and cessation of labor was a personal choice for the last three. Five subjects had professional training since TBI and it was a paid training for two of them. Five had vocational rehabilitation.

Of the 35 patients (42.7% of the 82 evaluated patients) who did not resume work, 31 (88.6%) declared that was because of TBI. Five retired after TBI or were on early retirement. None was a student or in vocational training at the time of evaluation. Six (17.1%) were currently searching for a job. Three (8.6%) were stay-at-home parents. Seven (20%) had an unpaid community-based and voluntary activity.

Univariate analysis of variables associated with return to work 8-year post injury are shown in Table 4. After corrections for multiple comparisons, a higher 1-year GOSE score, a lower patient's self-rating DEX score, independence in taking public transports, in finance and administrative management, and resuming of driving were significantly associated with return to work. Subjects who did not resume driving were more likely to be unemployed. There was a non-significant trend after correction for multiple comparison, for pre-injury occupational levels to impact return to work. Adjusted standardized residuals were calculated and showed that only unemployed subjects before TBI were more frequently unemployed 8 years after. There was also a trend for a lower initial GCS score, a longer length of stay in intensive care unit, a lower GOS score upon intensive care unit discharge, a higher HADS depression score, a higher DEX score assessed by a relative, swallowing difficulties, dependence in self-care activities, inability to write a letter to be associated with unemployment.

**Table 4:** Return to work. Univariate analysis.

**Patient characteristics**	**Unemployed (*n* = 39)**		**Employed (*n* = 37)**			
	**Mean ± SD [minimum; maximum] or count (%)**	**Missing data**	**Mean ± SD [minimum; maximum] or count (%)**	**Missing data**	**p (chi2) p (student)**	Corrected^*^ p (chi2) or p (student)
Gender		0 (0%)		0 (0%)	0.49	1
Femal	6 (15.4%)		9 (24.3%)			
Male	33 (84.6%)		28 (75.7%)			
Age (years)	33.8 ± 11.2 [15.4–52.4]	0 (0 %)	29.9 ± 10.8 [16.3–53.8]	0 (0 %)	0.124	1
Years of education	11.5 ± 3.3 [6–19]	3 (7.7 %)	12.5 ± 2.6 [7–17]	4 (10.8 %)	0.181	1
Occupational class		1 (2.6%)		1 (2.7%)	0.028	0.788
Blue collar	18 (47.4%)		13 (36.1%)			
White collar	7 (18.4%)		13 (36.1%)			
Student	7 (18.4%)		10 (27.8%)			
Unemployed	6 (15.8%)		0 (0%)			
Living alone before TBI		0 (0%)		0 (0%)	0.376	1
No	26 (66.7%)		29 (78.4%)			
Yes	13 (33.3%)		8 (21.6%)			
Alcohol abuse		4 (10.3%)		0 (0%)	0.321	1
No	31 (88.6%)		36 (97.3%)			
Yes	4 (11.4%)		1 (2.7%)			
GCS	5.3 ± 1.8 [3–8]	1 (2.6 %)	6.2 ± 1.7 [3–8]	0 (0 %)	0.039	0.97
Duration of coma (days)	10 ± 6.1 [0–23]	11 (28.2 %)	9.6 ± 6.2 [0–24]	3 (8.1 %)	0.84	1
Time to follow command (days)	13.2 ± 11.5 [0–56]	13 (33.3 %)	13 ± 10.5 [0–50]	5 (13.5 %)	0.946	1
Length of stay in ICU (days)	36.6 ± 28.2 [3–131]	0 (0 %)	24.2 ± 18.6[4–84]	0 (0 %)	0.026	0.766
GOS at ICU discharge	3.5 ± 0.8 [2–5]	8 (20.5 %)	3.9 ± 0.8 [3–5]	2 (5.4 %)	0.036	0.97
GOSE at 1 year	4.2 ± 1.1 [2–7]	15 (38.5 %)	5.5 ± 1.4 [4–8]	13 (35.1 %)	< 0.001	0.01
HADS anxiety score	6.7 ± 4.7 [0–17]	6 (15.4 %)	5.3 ± 4.4 [0–16]	0 (0 %)	0.193	1
HADS depression score	6.5 ± 4.8 [0–16]	6 (15.4 %)	4.2 ± 4.1 [0–14]	0 (0 %)	0.036	1
HADS total score	13.2 ± 8.1 [1–33]	6 (15.4 %)	9.4 ± 8.1 [0–27]	0 (0 %)	0.059	0.97
DEX score (patients)	21.8 ± 14.1 [3–59]	6 (15.4 %)	11.2 ± 10.7 [0–44]	0 (0 %)	0.001	0.029
DEX score (relatives)	30.2 ± 15.5 [3–71]	11 (28.2 %)	15.9 ± 17.4 [0–62]	23 (62.2 %)	0.015	0.459
Somatic and neurological complaints						
Headaches		5 (12.8%)		7 (18.9%)	0.921	1
No	23 (67.6%)		19 (63.3%)			
Yes	11 (32.4%)		11 (36.7%)			
Other pain		2 (5.1%)		0 (0%)	0.767	1
No	29 (78.4%)		31 (83.8%)			
Yes	8 (21.6%)		6 (16.2%)			
Motricity		2 (5.1%)		0 (0%)	0.097	1
No	18 (48.6%)		26 (70.3%)			
Yes	19 (51.4%)		11 (29.7%)			
Balance		2 (5.1%)		0 (0%)	0.161	1
No	17 (45.9%)		24 (64.9%)			
Yes	20 (54.1%)		13 (35.1%)			
Vision		2 (5.1%)		0 (0%)	0.132	1
No	22 (59.5%)		29 (78.4%)			
Yes	15 (40.5%)		8 (21.6%)			
Audition		2 (5.1%)		0 (0%)	0.734	1
No	31 (83.8%)		33 (89.2%)			
Yes	6 (16.2%)		4 (10.8%)			
Swallowing		1 (2.6%)		0 (0%)	0.037	0.97
No	30 (78.9%)		36 (97.3%)			
Yes	8 (21.1%)		1 (2.7%)			
Taste/smell		2 (5.1%)		0 (0%)	1	1
No	28 (75.7%)		27 (73%)			
Yes	9 (24.3%)		10 (27%)			
Lower urinary tract		2 (5.1%)		0 (0%)	0.155	1
No	30 (81.1%)		35 (94.6%)			
Yes	7 (18.9%)		2 (5.4%)			
Spasticity		2 (5.1%)		0 (0%)	0.615	1
No	24 (64.9%)		27 (73%)			
Yes	13 (35.1%)		10 (27%)			
Speech/Language		1 (2.6%)		0 (0%)	0.106	1
No	21 (55.3%)		28 (75.7%)			
Yes	17 (44.7%)		9 (24.3%)			
Independence						
Personal activities		0 (0%)		0 (0%)	0.039	0.97
No	6 (15.4%)		0 (0%)			
Yes	33 (84.6%)		37 (100%)			
Taking public transports		0 (0%)		0 (0%)	0.001	0.026
No	12 (30.8%)		0 (0%)			
Yes	27 (69.2%)		37 (100%)			
Writing a letter		0 (0%)		0 (0%)	0.012	0.366
No	15 (38.5%)		4 (10.8%)			
Yes	24 (61.5%)		33 (89.2%)			
Financial and administrative Management		0 (0%)		0 (0%)	< 0.001	0.002
No	25 (64.1%)		6 (16.2%)			
Yes	14 (35.9%)		31 (83.8%)			
Driving		0 (0%)		0 (0%)	< 0.001	0.001
No	23 (59%)		4 (10.8%)			
Yes	16 (41%)		33 (89.2%)			

TBI, Traumatic Brain Injury; GCS, Glascow Coma Scale; ICU, Intensive Care Unit; GOS, Glasgow Outcome Scale; GOSE, Glasgow Outcome Scale - Extended; HADS, Hospital Anxiety and Depression Scale; DEX, Dysexecutive Questionnaire; Personal activities: grooming, dressing, moving at home, using the bathroom.

* *Corrected p-value according to Holm*.

## Discussion

This is one of the first report of long-term outcome over 8 years in a sample of exclusively severe TBI included prospectively from the acute stage and from different emergency centers.

### Impairments, Activity Limitations, and Global Outcome

The main results were that very few, only 15% of the evaluated patients did not suffer somatic or neurological disability and most of them reported multiple and various complaints. Balance, motricity, and headaches were the most frequent complaints which corresponds with previous studies of patients recruited from a rehabilitation center (3). The very high rates of cognitive and behavioral problems reported in our sample and the high number of complaints per patient point out that they seemed more disabling than neurological or somatic disorders in the long-term. These results were in line with those reported 10 to 15 years after very severe TBI as defined by a post-traumatic amnesia duration of two months or more (32).

The probability of developing post-traumatic epilepsy increases with TBI severity (33). About 10% of the sample developed epilepsy and only one patient was not stabilized at 8-year follow-up. This result appeared to be quite similar to those described in previous reports with other TBI severity rating scales (3, 33, 34).

About a quarter of the sample had clinically significant anxiety or depression that seemed consistent with previous reports in long-term follow-up after rehabilitation (35). The management of stress and emotional disorders was among the most frequently perceived needs of patients in previous studies (36, 37).

Global outcome evaluated with GOSE score, revealed that about a third (28%) of the subjects were at a lower moderate disability level or severe disability, meaning they needed help for daily living activities. Another third (37%), were classified at an upper moderate disability level which means they were independent inside and outside the home but had a reduced work capacity, less but some social, and leisure activities or at some weekly family or friendship disruption. The last third (33%) obtained a good recovery according to the GOSE, which was in the upper range for 16%. These results were in line with previous reports with inclusion from acute care and comparable initial severity (38, 39) and worse than those described in studies including also mild and moderate TBI from rehabilitation units (35). In our study as in previous research, age and gender did not significantly influence the GOSE score at 8 years in the univariate analysis (38). However, in the multivariable analysis taking into account injury severity, older age was associated with poorer global long-term outcome as most frequently reported (4). A longer education duration was significantly associated with a better long-term global outcome even when taking into account initial severity in the multivariable model which was an original result not reported so far to our knowledge. Lower initial GCS score, longer length of stay in the intensive care unit, lower GOS score at intensive care unit discharge and lower GOSE score at 1 year were all significantly associated with lower GOSE score at 8 years. To our knowledge, the significant effect of these injury severity markers on long-term global functioning (as measured with GOSE score) in patients with severe TBI had rarely been found in previous research only including patients with severe TBI. Sigurdardottir et al. (40) found that initial TBI severity was significantly related to 1-year GOSE but they included patients with a much wider range of severity (including mild and moderate TBI)(40). Post-traumatic amnesia has been repeatedly found to be a significant predictor of outcome (3, 40–42). Unfortunately, post-traumatic amnesia was not available in a number of patients in our study and hence could not be included in our model. A higher DEX score rated by patients at 8 years was associated with a poorer global outcome even controlling for age and initial severity. This result confirmed the ecological validity of this scale in measuring cognitive and behavioral difficulties in patients with TBI (23).

Figure 6 shows that most of the patients were independent at home in accordance to GOSE results at 8 years. The independence rate decreased for tasks involving cognitive functions. This was consistent with the fact that cognitive complaints were the most common in these patients. Thus, only half of our sample was independent for financial and administrative management.

### Education and Work

All the students at the time of TBI who continued their studies after the trauma had a job at 8 years whereas seven of 11 who did not were unemployed. Academic achievement seemed a good predictor of the ability to work after TBI among students.

In our sample, almost half of the subjects under 65 had a job 8 years after TBI. In contrast, in an earlier study conducted 10 to 15 years after the trauma, 12.5% only had a job, but this was not a longitudinal study, and these patients presumably had a more severe TBI (32). In a more recent study, more than 50% of those studying or employed prior to injury returned to employment (3). In fact, rates reported in literature were very variable as some authors considered return to work and others employment, some included students and others not, TBI severity was not homogenous across studies, patients could be included from acute care or rehabilitation units, and the evaluation period ranged from 1 week to 23 years (42–55). Our results are probably representative of the whole population of patients with severe TBI, as it included patients prospectively followed-up from the day of the accident. However, a recent Nationwide follow-up study using weekly records on public assistance benefits in Denmark reported that only 30% returned to work after severe TBI and 16% achieving stable labor market attachment within 2 years (56).

Among patients with a productive employment, there were important changes in the characteristics of the job. About a quarter decreased their working time which was a slightly higher proportion than previously described (3). However, most of the patients (73%) worked 80% to full time. More than a third of workers reported an income decrease as previously described at 1 year post-TBI (57). On average, return to work occurred more than 2 years after TBI which was in line with previous research (56).

The only demographic or personal characteristic that impacted return to work in our study was the occupational class before TBI. Unemployment before the injury was the only demographic characteristic significantly associated with unemployment after TBI in accordance with previous research (4, 6). We did not find an effect of age on return to work at 8 years. Effect of age was not systematically reported in previous research (4). As in most studies gender was not associated with return to work (4). Length of education was not different between employed and unemployed patients in our sample in contrast with previous studies (6). As patients were more severe in our sample than in most of previous research, a possible explanation was that education duration might have a lower impact on the ability to work in severe TBI subjects than in mild to moderate ones. Regarding TBI severity, there was only a trend for patients with a lower initial GCS score, a shorter length of stay in ICU, a higher GOS score at ICU discharged to be unemployed at 8 years. In previous studies, rate of return to work decreased with TBI severity (6). In our study, because of the important number of evaluated factors, correction for multiple comparisons could have led to a false negative result of the effect of TBI severity on return to work. One year GOSE score was lower in unemployed patients in accordance with our previous findings on the same sample of patients at 4-year assessment (18). Among the variables measured at 8 years, none of the somatic or neurological complaints were associated with return to work. To the contrary, DEX scores assessed with patients was significantly higher in unemployed subjects. These results highlighted that employment on a long-term after TBI was more associated with behavioral than with somatic troubles. There was only a trend, non-significant after correction for multiple comparisons, for higher HADS depression score to be associated with unemployment. Only few previous studies reported a negative association between return to work and depression (58, 59), most of these found no significant association (60). Personality changes have been found significantly associated with unemployment 18 months after severe TBI (61), in accordance with the present results at a longer term post-injury. Finally, not surprisingly, independence in personal care, the ability to use public transport, and to manage administrative duties were all significantly associated with employment.

### Limitations

Our study has several limitations. First, a part of the collected data was reported by patients or relatives, and some measures were not obtained for the whole sample because of time required to collect this important amount of data. The second limitation is the high rate of lost to follow-up which is frequent in this kind of long-term follow-up studies (9, 40, 62–64). However, patients lost to follow-up or those who refused to participate only significantly differed regarding pre-TBI occupational status. Hence, our sample seemed representative of the original cohort. However, we could not exclude bias as previous work showed that socially disadvantaged persons were underrepresented in TBI outcome research (22). To avoid this bias and improve the knowledge of TBI outcome and its predictors, future works based on data from national or regional registers as in recent work of Odgaard et al. (56) and confrontation with the initial TBI data would be helpful.

## Conclusion

We provided a rare comprehensive description of long-term outcome in an inception cohort of exclusively severe TBI patients included at the acute stage. Most important results were the low rate of full recovery on a long-term, the high rates of complaints and particularly those concerning cognitive and behavioral disorders. The long-term global outcome was related to age, education duration, initial injury severity, and persistent dysexecutive syndrome. Return to work remained relatively low and those who had a job still experienced a number of difficulties at work, raising concerns about job stability. Dysexecutive disorders had a significant impact on long-term employment. These results reinforced the importance of long-term follow-up in patients with severe TBI and the need for specific interventions mainly aimed at the management of cognitive disorders and socio-professional reintegration.

## Data Availability

The datasets generated for this study are available on request to the corresponding author.

## Author Contributions

PAz, CJ, EB, PP-D, AR, and JC contributed to the conception and design of the study. GN collected data. IG organized the database and performed the statistical analysis. AR performed the statistical analysis and wrote the first draft of the manuscript. PAz, CJ, EB, AL, and CV-A critically revised the draft and provided updates. All authors contributed to the manuscript revision, read, and approved the submitted version.

### Conflict of Interest Statement

The authors declare that the research was conducted in the absence of any commercial or financial relationships that could be construed as a potential conflict of interest.
